# Troponin level as a predictor of prognosis in patients with acute ischemic stroke

**DOI:** 10.1186/cc14549

**Published:** 2015-03-16

**Authors:** H Bayir, R Dagli, H Kaymaz, I Yildiz, H Kocoglu

**Affiliations:** 1 Abant Izzet Baysal University, Medical School, Bolu, Turkey; 2 Ahi Evran University Education and Research Hospital, Kirsehir, Turkey

## Introduction

The aim of this study was to identify the association between troponin level and the outcome in patients with acute ischemic stroke.

## Methods

We retrospectively investigated 152 patients admitted to our reanimation ICU for cerebrovascular accident between 1 January 2013 and 31 December 2013. Inclusion criteria were as follows: patients with acute ischemic stroke, measurement of serum troponin level and electrocardiography performed within 24 hours of admission. Not included were patients with intracerebral hemorrhage, no brain imaging or electrocardiography, previous myocardial infarction, stable or unstable angina pectoris before admission, previous coronary angioplasty or coronary bypass surgery.

## Results

**Figure 1:**
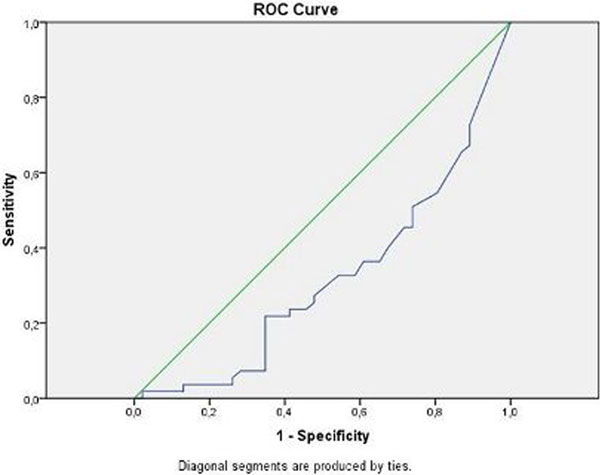


Of 152 patients, 51 patients were excluded from the study because of the exclusion criteria. The serum troponin level was elevated in 81 patients. The patients were divided into two groups; patients in group 1 (*n *= 81) with serum troponin level >0.01, and those in group 2 (*n *= 20) with serum troponin level ≤0.01. For 1-month follow-up results of patients, death had occurred in 50.6% (*n *= 41) of patients in group 1 and in 25% (*n *= 5) of patients in group 2. There was a significant positive correlation between the increase in troponin level and death within 1 month (*r *= 0.205; *P *= 0.040). The best cutoff point revealed by the ROC curve of troponin was 0.291 mg/l; at which the sensitivity was 73% and the specificity was 79% when used for prediction of death within 1 month (area = 0.319, CI = 0.214 to 0.423, *P *= 0.021; Figure 1).

## Conclusion

These results suggest that increased serum troponin level at admission is associated with higher mortality rate. Troponin positivity on admission is an independent prognostic predictor in acute ischemic stroke.
